# Inhaled nitric oxide in moderate-to-severe COVID-19 acute respiratory distress syndrome: a retrospective cohort study

**DOI:** 10.31744/einstein_journal/2024AO0578

**Published:** 2024-07-29

**Authors:** Lucas Eduardo Benthien Santos, Camila Campos Grisa Padovese, Isabela Belarmino Oliveira de Castro, Rodrigo Carneiro Franco, Ana Paula Pires Bolsoni Okuda, Mariana Resende Bustamante, Luciana Gioli-Pereira

**Affiliations:** 1 Hospital Municipal da Vila Santa Catarina Dr. Gilson de Cássia Marques de Carvalho Hospital Israelita Albert Einstein São Paulo SP Brazil Hospital Municipal da Vila Santa Catarina Dr. Gilson de Cássia Marques de Carvalho; Hospital Israelita Albert Einstein, São Paulo, SP, Brazil.

**Keywords:** COVID-19, Coronavirus infections, SARS-CoV-2, Nitric oxide, Respiratory distress syndrome, Length of stay, Critical illness, Intensive care units

## Abstract

**Objective:**

To evaluate the responsiveness of COVID-19 acute respiratory distress syndrome patients to inhaled nitric oxide as part of their standard therapy.

**Methods:**

This retrospective cohort study included critically ill adult patients with confirmed COVID-19 treated between March 2020 and May 2021. Eligible patients with moderate-to-severe acute respiratory distress syndrome due to COVID-19 were subsequently categorized into two groups based on inhaled nitric oxide use throughout their stay in the intensive care unit. The primary endpoints were overall mortality and improvement in oxygenation parameters 6 hours after inhaled nitric oxide use.

**Results:**

A total of 481 patients admitted to the intensive care unit due to COVID-19 acute respiratory distress syndrome were screened, 105 of which were included. Among the 105 patients, inhaled nitric oxide therapy was used in 33 patients, will 72 did not undergo inhaled nitric oxide therapy. No significant difference in mortality was observed between the groups (67% for the treatment and 82% for the no-treatment groups respectively, p=0.173). Among the patients who used inhaled nitric oxide, 17 (51%) were considered responsive to therapy. There was no significant difference in the length of stay in the intensive care unit (p=0.324) or total hospitalization time (p=0.344).

**Conclusion:**

Inhaled nitric oxide rescue therapy improved oxygenation in patients with COVID-19 with moderate-to-severe acute respiratory distress syndrome but did not affect mortality.

## In Brief

In this study, we present the findings from a cohort of patients with COVID-19 with acute respiratory distress syndrome who underwent standard therapy, including prone positioning, with or without adjunctive inhalation of nitric oxide. Our investigation sought to determine whether inhaled nitric oxide administration yielded clinical enhancement in this population. Remarkably, nitric oxide administration elevated the PaO2/FiO2 ratio, which is indicative of improved oxygenation. Despite this improvement, discernible mortality benefits did not emerge in association with the inhaled nitric oxide treatment.

## Highlights

Acute respiratory distress syndrome is a severe presentation of COVID-19 without effective treatment despite intensive research.

Inhaled nitric oxide is a vasodilator that has been studied in patients with acute respiratory distress syndrome COVID-19 yielding contradictory results.

The use of inhaled nitric oxide rescue therapy in critically ill patients with COVID-19 experiencing moderate-to-severe acute respiratory distress syndrome was significantly associated with an improvement in oxygenation parameters with no difference in mortality.

## Graphical abstract

**Figure:**
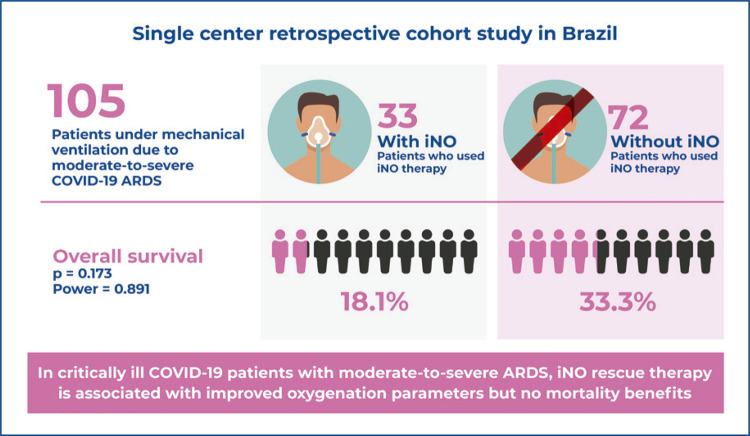

## INTRODUCTION

Acute respiratory distress syndrome (ARDS) is a severe presentation of COVID-19, the disease caused by SARS-CoV-2.^(1)^ Despite intensive research, some treatments have demonstrated certain effects, although none can be considered excellent for treating COVID-19-induced ARDS.^(2)^

During this pandemic, intensive research was conducted to identify therapeutic options. Among the studied medications is nitric oxide (NO), an endogenous signaling molecule that can be inhaled. Prior to the pandemic, inhaled nitric oxide (iNO) had been tested as a possible therapeutic option for ARDS.^(3-4)^ A meta-analysis published by Cochrane in 2016 showed an improvement in the PaO_2_/FiO_2_ ratio in ARDS of any cause with iNO, although no impact on mortality was observed.^(4)^

Since the advent of COVID-19, NO has emerged as a potential therapeutic target. Physiologically, NO has bronchodilatory, antithrombotic, and arterial vasodilatory effects.^(5)^ Akaberi et al.^(6)^ showed that NO, *in vitro,* also has an anti-replicative effect against SARS-CoV-2. Therefore, NO was proposed to potentially improve oxygenation and outcomes in critically ill patients with ARDS arising from COVID-19.

There are few publications on the effectiveness of iNO in the treatment of ARDS caused by COVID-19, and they have controversial results. Two series of cases with a small number of patients showed little response to this therapy.^(7,8)^ Contrary to these findings, one clinical trial and one retrospective cohort of 71 patients reported a response rate of approximately two-thirds when considered improvement by >20% in terms of the PaO_2_/FiO_2_ ratio.^(9,10)^Recently, a multicenter cohort study with an iNO Group of 76 patients found an improvement in oxygenation parameters; however, there was no difference in mortality rates.^(11)^ In addition, a recent meta-analysis that included eight studies and 265 patients found an improvement in oxygenation parameters in 66% of the patients, but without benefits to the mortality rate.^(12)^

The combined use of iNO with other pulmonary vasoactive agents, such as almitrine, has been studied. Almitrine has been reported to elevate oxygenation in patients with ARDS owing to its hypoxic pulmonary vasoconstriction action. Some studies have evaluated the NO associated with almitrine, with results suggesting a possible therapeutic benefit of this combination.^(13-16)^

This study is an observational retrospective medical chart review conducted at a public COVID-19 reference center in São Paulo, where iNO was used as a rescue therapy in a group of mechanically ventilated patients with moderate-to-severe COVID-19 ARDS. We hypothesized that iNO could improve oxygenation parameters and reduce mortality in patients with COVID-19 and ARDS.

## OBJECTIVE

To evaluate the responsiveness of COVID-19 acute respiratory distress syndrome patients to inhaled nitric oxide as part of their standard therapy. We compared all-cause mortality and the duration of mechanical ventilation between patients who inhaled or did not inhale nitric oxide.

## METHODS

This study was conducted in accordance with the Declaration of Helsinki. The requirement for informed consent was waived by the Research Ethics Committee of *Hospital Israelita Albert Einstein* owing to the retrospective, non-interventional nature of the study and anonymized data analysis. The study was approved under the registration numbers (CAAE: 53009721.0.0000.0071; #5.200.505. Data analysis was conducted in an aggregated manner so that the secrecy and privacy of the data were respected throughout the process.

### Study design

This study was a retrospective medical chart review conducted at a single center, a public hospital that served as a reference for the treatment of COVID-19 in São Paulo, Brazil, during the pandemic. The period analyzed was from March 2020 to May 2021.

### Inclusion and exclusion criteria

Adult patients aged >18 years with a COVID-19 diagnosis and admitted to the intensive care unit (ICU) were screened for enrollment. All patients under mechanical ventilation for moderate-to-severe COVID-19 ARDS and who were placed in the prone position and/or received iNO were included.

Patients with no confirmed diagnosis of COVID-19 or those who did not require mechanical ventilation were excluded. Patients admitted to the ICU for reasons other than ARDS, and those with incomplete information in their medical records were excluded. In this study ARDS was defined according to the American-European Consensus Conference (AECC),^(17,18)^ and COVID-19 was considered the cause when confirmed by RT-PCR.

### Intervention

As standard institutional therapy, all patients were ventilated under protective parameters for 6 hours, and the PaO_2_/FiO_2_ ratio was measured. If the ratio was <200, a PEEP titration was performed, and 2 hours later, a new blood gas analysis was performed. If the PaO_2_/FiO_2_ ratio was <150 after PEEP titration, the prone position was indicated, and the ratio was evaluated 4 hours later. Patients who evolved with an increase of PaO_2_/FiO_2_ ≥20 after the prone position were maintained in the position for 16 to 20 hours were considered responsive to therapy. Those who did not present with an increase in PaO_2_/FiO_2_ ≥20 were considered not responsive and were treated with alveolar recruitment in the prone position. In cases where there was no response to the prone position or when it could not be applied, iNO was considered as a rescue therapy. Inhaled NO was administered for a minimum period of 6 hours until ventilatory improvement was achieved, which was measured using the PaO_2_/FiO_2_ ratio. We utilized the iNO dose institutionally applied in clinical practice (20 parts per million), and the gas was reduced or suspended based on signs of toxicity or suspicion thereof.

We analyzed PaO_2_/FiO_2_ before and after prone positioning, as well as with iNO therapy. We considered responders to be patients with a 20% increase in the PaO_2_/FiO_2_ ratio in the first 2 hours after the start of iNO therapy.^(7-10,12,13)^

All patients received dexamethasone as standard glucocorticoid therapy, and there were no additional medications intended to treat COVID-19. Other medications such as sedatives, neuromuscular blockers, inotropes, vasoconstrictors, antibiotics, anticoagulants, and antiarrhythmics were used according to the patient’s clinical needs.

### Data collection

Demographics, clinical data, and complementary examinations were collected from all the patients included in the study. The patients were divided into two groups: with and without iNO therapy. These groups were matched by age, sex, PaO_2_/FiO_2_ ratio, and comorbidities. The responsiveness of the patients who received iNO was evaluated according to previous reports.^(7-10,12,13)^

The primary endpoints analyzed were responsiveness to iNO and all-cause mortality during ICU stay. The secondary endpoints were the time of mechanical ventilation and length of ICU stay.

### Statistical analysis

Data were described as mean, standard deviation, minimum, maximum, median, and quartiles for quantitative variables. Frequency tables were used for the qualitative variables. To compare the clinical outcomes between the groups, the χ^2^ or Fisher’s exact test and Student’s *t*-test or– the Mann-Whitney test were used, depending on the characteristics of the distribution.

Non-parametric Kaplan-Meier analyses were performed to estimate the overall survival of patients in the ICU and after application of the prone position and iNO treatment. The log-rank test was used to compare the treatment survival curves. Patient survival was censored based on the date of ICU discharge.

The normality of the variables was checked using the Shapiro-Wilk test. The significance level adopted in these analyses was set at 5%. A power test was performed, and the results are presented in a table. Analyses were performed using R software, version 4.1.1.

## RESULTS

**Figure 1:**
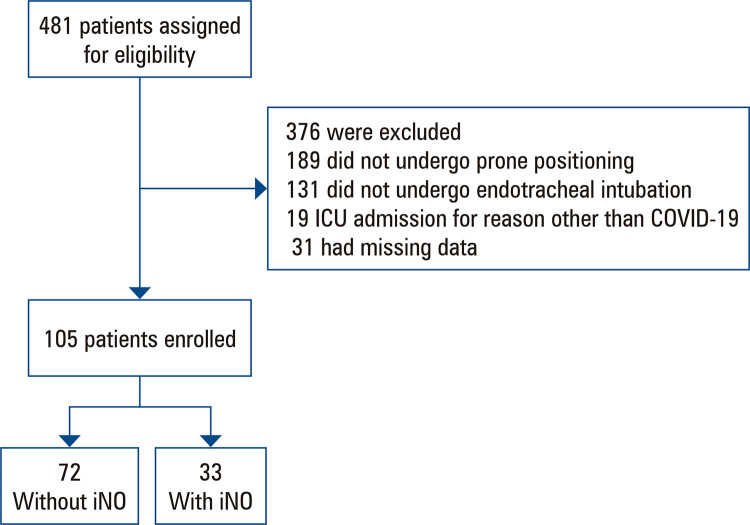
Recruitment process of COVID-19 patients screened for eligibility

A total of 481 patients with COVID-19 and ARDS admitted to the ICU were screened for enrollment, and 105 were included in the study. The patients were divided into two groups: patients who received iNO therapy (33) and those who did not (72) (Figure 1).

ICU: intensive care unit.

The baseline characteristics of the individuals were male predominance (60%) and a median age of 56 years. The main comorbidities in overall patients were hypertension, *diabetes mellitus* and obesity. Alcohol consumption differed between groups (p=0.014; Table 1).

**Table 1:** Demographics and baseline characteristics of the groups without and with inhaled nitrogen oxide therapy

Variable	Without iNO Group (n=72)	With iNO Group (n=33)	p value
Age median (year)	62 [49.0–68.2]	56 [45.0–63.0]	0.056
Sex	72 (100)	33 (100)	0.324
Female	26 (36.1)	16 (48.5)	
Male	46 (63.8)	17 (51.5)	
Smoking	7 (9.7)	5 (15.1)	0.630
Alcohol	0 (0)	4 (12.1)	0.014
Obesity	29 (40.2)	8 (24.2)	0.169
Hypertension	42 (58.3)	17 (51.5)	0.659
Diabetes	26 (36.1)	14 (42.4)	0.688
COPD	4 (5.5)	3 (9.0)	0.800
Asthma	3 (4.1)	2 (6.0)	0.999
Coronary artery disease	2 (2.7)	2 (6.0)	0.790
Heart Failure	4 (5.5)	1 (3.0)	0.944
Chronic kidney disease	4 (5.5)	2 (6.0)	0.999
Cancer	7 (9.7)	6 (18.1)	0.367
Solid-organ transplant	9 (12.5)	8 (24.24)	0.218
Hypothyroidism	7 (9.72)	2 (6.06)	0.805

Values are presented as median [interquartile range] or n (%). χ^2^ test (significance level was 5%).

COPD: chronic obstructive pulmonary disease; iNO: inhaled nitric oxide.

The responsiveness to the prone position was higher in the group that did not use iNO (p=0.005, power=0.999). In addition, the PaO_2_/FiO_2_ ratio after prone positioning was higher in the group without iNO (p=0.008, power=0.890; Table 2). There were no differences between the groups regarding the other patterns of intensive support care (Table 3).

**Table 2:** Prone position response of groups without and with inhaled nitric oxide therapy

	Without iNO Group	With iNO Group	p value
Prone	72 (100)*	24 (72.7)*	<0.001
Effective response	59 (81.9)*	12 (50)*	0.005
PaO_2_/FiO_2_ ratio			
Before prone	108±24.9^†^	115.7±26.6^†^	0.225
After prone	179.5 [132.8-238.5] ^‡^	142.5 [107.0-186.2] ^‡^	0.008

Values are presented as n (%), mean±standard deviation, or median [interquartile range].

* χ^2^ test (significance level was 5%); ^†^ Student *t*-test (significance level was 5%); ^‡^ Mann-Whitney test (significance level was 5%).

iNO: inhaled nitric oxide.

**Table 3:** Intensive care support for groups without and with inhaled nitric oxide therapy

Intensive care support	Without iNO Group (n=72)	With iNO Group (n=33)	p value
Hemodialysis	44 (61.1)	17 (51.5)	0.476
Dobutamine	2 (2.7)	4 (12.1)	0.144
Amiodarone	14 (19.4)	8 (24.2)	0.762
Anticoagulation	28 (38.8)	19 (57.5)	0.115
Vasoconstrictor agents	58 (80.5)	26 (78.7)	0.999
Tracheostomy	16 (22.2)	10 (30.3)	0.518

Values are presented as n (%).

χ^2^ test (significance level was 5%).

iNO: inhaled nitric oxide.

**Figure 2:**
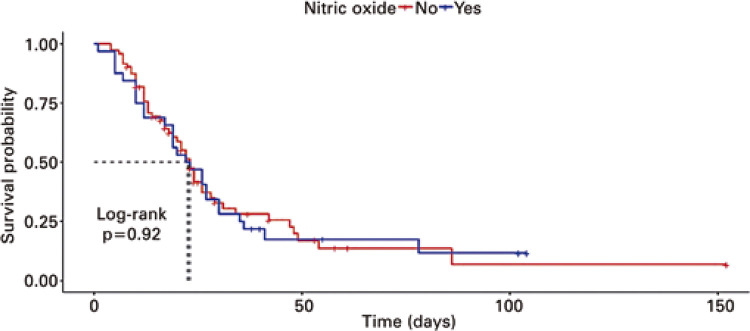
Survival analysis comparison between groups without and with inhaled nitric oxide therapy

The primary endpoints were iNO responsiveness and overall mortality during the ICU stay. There was no significant difference in the overall mortality between the groups (p=0.173, power=0.891; Table 4 and Figure 2). Of the 33 patients who received iNO, 9 had not previously undergone prone positioning due to contraindications such as hemodynamic instability and body mass index over 40kg/m^2^. Among patients who used iNO, 17 (51%) were considered responsive to therapy. Regarding secondary outcomes, we did not observe differences in mechanical ventilation time (p=0.383), ICU length of stay (p=0.324), or total hospitalization time (p=0.344; Table 4).

**Table 4:** Patient outcomes for patients with COVID-19 induced acute respiratory distress syndrome who received and did not receive inhaled nitric oxide therapy

	Without iNO Group (n=72)	With iNO Group (n=33)	p value	Power
*Death	48 (66.6)	27 (81.8)	0.173	0.891
Discharged alive from the ICU*	24 (33.3)	6 (18.1)		
Mechanical ventilation (days) ^†^	14 [9.0–27.0]	19 [7.8–34.8]	0.383	0.164
Hospital LOS (days) ^†^	22 [12.8–33.2]	27 [14.0–41.0]	0.344	0.175
ICU LOS (days) ^†^	18 [10.8–26.0]	22 [11.5–35.2]	0.324	0.192

Values are presented as n (%) and median [interquartile range].

* χ^2^ test (significance level was 5%); ^†^ Mann-Whitney test (significance level was 5%).

ICU: intensive care unit; LOS: length of stay; iNO: inhaled nitric oxide.

The median survival of the group without iNO was 20 days (95% confidence interval (95%CI) =7 – missing value days), whereas that of patients in the iNO Group was 23 days (95%CI=19 – 27 days).

## DISCUSSION

We studied a cohort of patients with COVID-19-induced ARDS treated with standard therapy, including those in the prone position with or without iNO. We hypothesized that iNO would improve oxygenation parameters and reduce mortality rates in these patients. We did not observe a mortality benefit despite 51% of the patients in this study using iNO showing improved PaO_2_/FiO_2_ ratios.

Literature on the use of iNO in ARDS caused by COVID-19 is still scarce, but previous studies have shown similar responsiveness to iNO. For instance, Abou-Arab et al.^(9)^ conducted a clinical trial involving 34 patients and reported a response rate of 65%. Abman et al.^(10)^ reported a response rate of 62% in a retrospective cohort of 37 patients treated with iNO. In a recent meta-analysis, Alqahtani et al.^(12)^ observed an overall response rate of 66% among the seven studies included but with considerable heterogeneity between the results of the studies analyzed. Other studies with smaller sample sizes observed lower responses to iNO than those mentioned above.^(7-8)^

In fact, not only is responsiveness to iNO controversial among the few reports, but so is the methodology used. While Abou-Arab et al.^(9)^ used NO before indicating pronation and only performed pronation if there was no response, we used NO as a rescue therapy. Furthermore, Abman et al.^(10)^ used iNO in patients with a PaO_2_/FiO_2_ ratio between 100 and 300, which represents a less severe spectrum of ARDS than that observed in our study population with moderate-to-severe levels.

Another recent multicenter cohort study included 815 patients and evaluated the use of iNO in 76 of them. In agreement with most previous reports, including ours, they observed an improvement in oxygenation parameters but without a difference in mortality rates.^(11)^ Considering adverse effects with the iNO use, Al Sulaiman et al.^(11)^ described a higher incidence of acute kidney injury, pneumonia and acute liver injury associated with the use of iNO. No differences in renal impairment or dialysis indications were observed between the groups.

Despite a 51% response rate to iNO in our cohort, we did not observe any mortality benefits. These results are similar to those of previous studies, but they should be interpreted with caution because of the non-randomized design of the study. Although we followed a similar methodological design as in previous observational trials, we believe that the retrospective and non-randomized nature of the study may have interfered with the measurement of the effects of iNO. In our sample, NO was used in the most severe and refractory patients in the prone position as a rescue therapy. As a result, we believe that there was a selection bias, which we were able to identify when a significant difference was found in the post-prone PaO_2_/FiO_2_ ratio between the groups, suggesting worse basal oxygenation in the iNO Group. The design of a randomized clinical trial with matched control and treatment groups should follow as the next step in further studies.

### LIMITATIONS

This study had several limitations. First, this was a retrospective observational study; therefore, there was no planned design for the matched groups. Secondly, the sample size was small. Finally, we were unable to eliminate the selection bias of the most severely ill patients receiving iNO therapy, which could have minimized a possible unmeasured effect. However, this was a hypothesis-generating study, and randomized clinical trials can provide stronger evidence.

## CONCLUSION

We conclude that inhaled nitric oxide rescue therapy in COVID-19 patients with moderate-to-severe acute respiratory distress syndrome is associated with an augmentation in oxygenation with no mortality benefits. Neither mechanical ventilation nor intensive care unit length of stay were altered as a result of inhaled nitric oxide therapy. Further randomized clinical studies are necessary to confirm these findings.

## References

[1] Wu Z, McGoogan JM (2020). Characteristics of and Important lessons from the coronavirus disease 2019 (COVID-19) outbreak in China: Summary of a report of 72 314 cases from the chinese center for disease control and prevention. JAMA.

[2] World Health Organization (WHO) (2022). Weekly epidemiological update on COVID-19.

[3] Chen L, Liu P, Gao H, Sun B, Chao D, Wang F (2004). Inhalation of nitric oxide in the treatment of severe acute respiratory syndrome: a rescue trial in Beijing. Clin Infect Dis.

[4] Gebistorf F, Karam O, Wetterslev J, Afshari A (2016). Inhaled nitric oxide for acute respiratory distress syndrome (ARDS) in children and adults. Cochrane Database Syst Rev.

[5] Jin RC, Loscalzo J (2010). Vascular nitric oxide: formation and function. J Blood Med.

[6] Akaberi D, Krambrich J, Ling J, Luni C, Hedenstierna G, Järhult JD (2020). Mitigation of the replication of SARS-CoV-2 by nitric oxide in vitro. Redox Biol.

[7] Tavazzi G, Pozzi M, Mongodi S, Dammassa V, Romito G, Mojoli F (2020). Inhaled nitric oxide in patients admitted to intensive care unit with COVID-19 pneumonia. Crit Care.

[8] Ferrari M, Santini A, Protti A, Andreis DT, Iapichino G, Castellani G (2020). Inhaled nitric oxide in mechanically ventilated patients with COVID-19. J Crit Care.

[9] Abou-Arab O, Huette P, Debouvries F, Dupont H, Jounieaux V, Mahjoub Y (2020). Inhaled nitric oxide for critically ill Covid-19 patients: a prospective study. Crit Care.

[10] Abman SH, Fox NR, Malik MI, Kelkar SS, Corman SL, Rege S (2022). Real-world use of inhaled nitric oxide therapy in patients with COVID-19 and mild-to-moderate acute respiratory distress syndrome. Drugs Context.

[11] Al Sulaiman K, Korayem GB, Altebainawi AF, Al Harbi S, Alissa A, Alharthi A (2022). Evaluation of inhaled nitric oxide (iNO) treatment for moderate-to-severe ARDS in critically ill patients with COVID-19: a multicenter cohort study. Crit Care.

[12] Alqahtani JS, Aldhahir AM, Al Ghamdi SS, AlBahrani S, AlDraiwiesh IA, Alqarni AA (2022). Inhaled Nitric Oxide for Clinical Management of COVID-19: a Systematic Review and Meta-Analysis. Int J Environ Res Public Health.

[13] Caplan M, Goutay J, Bignon A, Jaillette E, Favory R, Mathieu D, Parmentier-Decrucq E, Poissy J, Duburcq T, Lille Intensive Care COVID-19 Group (2021). Almitrine Infusion in Severe Acute Respiratory Syndrome Coronavirus 2-Induced Acute Respiratory Distress Syndrome: a Single-Center Observational Study. Crit Care Med.

[14] Bagate F, Tuffet S, Masi P, Perier F, Razazi K, de Prost N (2020). Rescue therapy with inhaled nitric oxide and almitrine in COVID-19 patients with severe acute respiratory distress syndrome. Ann Intensive Care.

[15] Laghlam D, Rahoual G, Malvy J, Estagnasié P, Brusset A, Squara P (2021). Use of Almitrine and Inhaled Nitric Oxide in ARDS Due to COVID-19. Front Med (Lausanne).

[16] Cardinale M, Esnault P, Cotte J, Cungi PJ, Goutorbe P (2020). Effect of almitrine bismesylate and inhaled nitric oxide on oxygenation in COVID-19 acute respiratory distress syndrome. Anaesth Crit Care Pain Med.

[17] Raghavendran K, Napolitano LM (2011). Definition of ALI/ARDS. Crit Care Clin.

[18] Bernard GR, Artigas A, Brigham KL, Carlet J, Falke K, Hudson L (1994). The American-European Consensus Conference on ARDS. Definitions, mechanisms, relevant outcomes, and clinical trial coordination. Am J Respir Crit Care Med.

